# Supplementary material for: Multifaceted information-seeking motives in children
Source: Nat Commun. 2023 Sep 7;14:5505. doi: 10.1038/s41467-023-40971-x (PMC10485006; doi:10.1038/s41467-023-40971-x)
Supplement: Supplementary file 1 — Supplementary Information [file 41467_2023_40971_MOESM1_ESM.pdf]

Supplementary Information

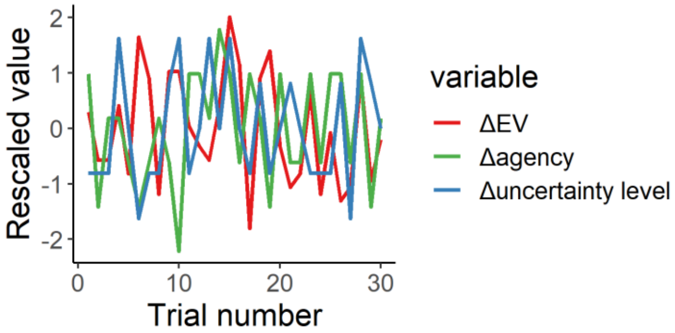

Supplementary Figure 1. Fluctuations of ΔEV, Δagency and Δuncertainty for an example participant. Z-scored values of ΔEV, Δagency, and Δuncertainty for an example participant varied independently of each other.

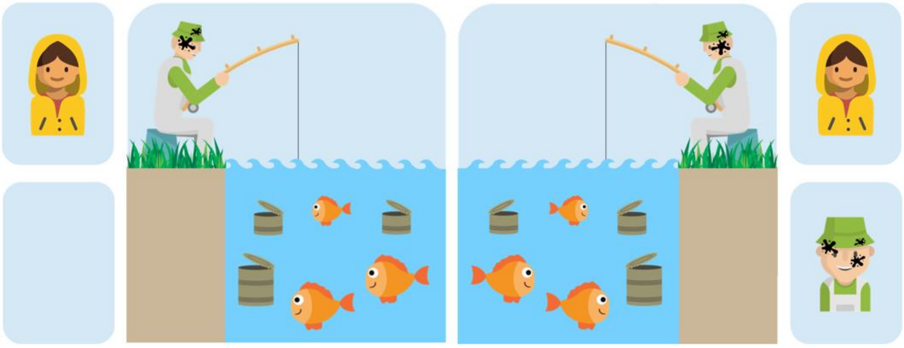

Supplementary Figure 2. Example trial from Experiment 3. Agency probability was always 100% (only the participant's icon was shown) in one pond and 50% (both the fisherman and the participants' icon were shown) in the other. The ponds were otherwise identical. Fisherman icon: Flaticon.com.

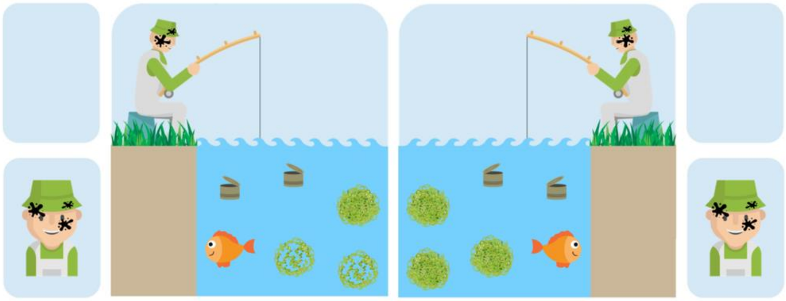

Supplementary Figure 3. Example trial from Experiment 4. Ponds could include both "seaweed with holes" ("empty seaweed"), with nothing hiding behind them, and "full seaweed", behind which fish or cans can be hiding. In this example, uncertainty was greater on the right pond due to more "full seaweed". Fisherman icon: Flaticon.com.

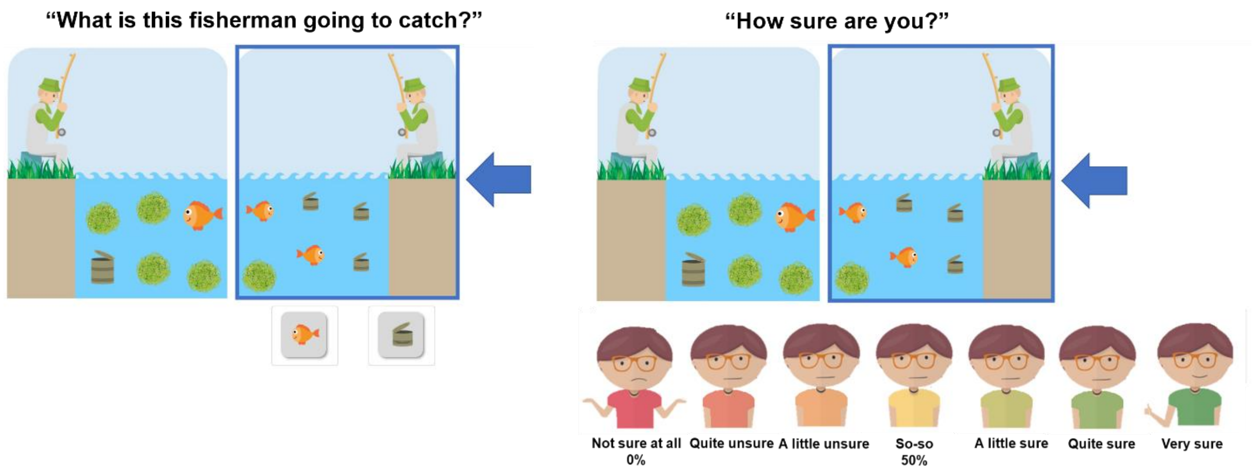

Supplementary Figure 4. Example trial for Experiment 5. After being shown the ponds for 4s, participants were asked (for each pond) whether they thought the respective fisherman would catch a fish or a can and how sure they were. Note that the uncertainty scale shown above is magnified for legibility.

Variable	β		S.E.M.		X^2		*p*		ω	
	Exp. 1	Exp. 2	Exp. 1	Exp. 2	Exp. 1	Exp. 2	Exp. 1	Exp. 2	Exp. 1	Exp. 2
ΔEV	0.59	0.66	0.07	0.07	69.22	81.87	<0.001	<0.001	0.58	0.65
Δagency	0.9	0.67	0.08	0.06	129.04	121.07	<0.001	<0.001	0.80	0.79
Δuncertainty	0.26	0.3	0.04	0.05	39.28	43.44	<0.001	<0.001	0.44	0.47
Age	0.19	-0.03	0.07	0.06	8.36	0.3	0.004	0.584	0.20	0.05
Previous choice	0.01	-0.05	0.07	0.06	0.04	0.48	0.851	0.487	0.01	0.04
Instructions comprehension	0.02	-0.02	0.06	0.05	0.17	0.14	0.68	0.709	0.03	0.03
EV comprehension	-0.1	0.12	0.06	0.05	3.45	6.9	0.063	0.009	0.13	0.19
Correct fishing	0	0.07	0.06	0.05	0	2.29	0.958	0.13	0	0.11
ΔEV * Age	0.11	0.03	0.07	0.07	3.01	0.16	0.083	0.686	0.12	0.03
Δagency * Age	0.34	0.16	0.08	0.06	23.92	9.03	<0.001	0.003	0.34	0.21
Δuncertainty * Age	0.2	0.13	0.04	0.05	23.96	9.27	<0.001	0.002	0.34	0.22
Previous choice * Age	-0.19	-0.09	0.06	0.06	9.51	2.06	0.002	0.151	0.22	0.10

Supplementary Table 1. A simple heuristic cannot explain interactions between information-seeking motives and age. Model results from a logistic mixed-effects regression predicting information-seeking in children in Experiments 1 and 2. The model was identical to the one described in the main text, except for the addition of information-seeking choices on the previous trial (0 for left, 1 for right), and the interaction between this factor and age. Therefore, the model had random intercepts for each participant, fixed effects and random slopes for ΔEV, Δagency, and Δuncertainty, and fixed effects for age and its interaction with each Δ and the previous choice, for instructions comprehension scores, EV comprehension scores, and proportion of correct fishing choices. As in the main analyses, we found significant effects of ΔEV, Δagency, and Δuncertainty. The interactions between age and Δagency, and between age and Δuncertainty remained significant even after accounting for information-seeking choices in the previous trial.

Variable	β		S.E.M.		X^2		p		ω	
	Exp. 1	Exp. 2	Exp. 1	Exp. 2	Exp. 1	Exp. 2	Exp. 1	Exp. 2	Exp. 1	Exp. 2
ΔEV	0.59	0.67	0.07	0.07	70.56	82.67	<0.001	<0.001	0.59	0.65
Δagency	0.90	0.67	0.08	0.06	127.93	122.03	<0.001	<0.001	0.76	0.79
Δuncertainty	0.27	0.31	0.04	0.05	41.62	46.76	<0.001	<0.001	0.45	0.49
Age	0.09	-0.06	0.06	0.05	2.40	2.03	0.121	0.154	0.11	0.10
Instructions comprehension	0	-0.02	0.06	0.05	0.01	0.19	0.94	0.663	0.01	0.03
EV comprehension	-0.09	0.12	0.06	0.05	3.04	7.37	0.081	0.007	0.12	0.19
Correct fishing	0	0.07	0.06	0.05	0	2.15	1	0.142	0	0.10
ΔEV * Age	0.11	0.02	0.07	0.07	2.82	0.13	0.093	0.718	0.12	0.03
Δagency * Age	0.34	0.17	0.08	0.06	23.89	10.12	<0.001	0.001	0.34	0.23
Δuncertainty * Age	0.2	0.14	0.04	0.04	24.55	10.37	<0.001	0.001	0.35	0.23
ΔEV * Δagency * Age	0.06	0.04	0.04	0.04	2.50	1.01	0.114	0.315	0.09	0.07
ΔEV * Δuncertainty * Age	-0.04	0.03	0.03	0.03	1.79	1	0.181	0.317	0.11	0.07
Δagency * Δuncertainty * Age	-0.01	0.01	0.04	0.03	0.07	0.18	0.795	0.668	0.02	0.03

Supplementary Table 2. Triple interactions between information-seeking motives and age were not significant and did not alter the results. Model results from a logistic mixed-effects regression predicting information-seeking in children in Experiments 1 and 2. The model was identical to the one described in the main text, except for the addition of triple interactions between each pair of motives (ΔEV, Δagency, and Δagency) and age. Therefore, the model had random intercepts for each participant, fixed effects and random slopes for ΔEV, Δagency, and Δuncertainty, and fixed effects for age and its interaction with each Δ as well as with pairs of Δs, for instructions comprehension scores, EV comprehension scores, and proportion of correct fishing choices. As in the main analyses, we found significant effects of ΔEV, Δagency, and Δuncertainty. Importantly, the interactions between age and Δagency, and between age and Δuncertainty remained significant even after accounting for additional triple interactions, none of which were significant.

Variable	β		S.E.M.		X^2		p		ω	
	Exp. 1	Exp. 2	Exp. 1	Exp. 2	Exp. 1	Exp. 2	Exp. 1	Exp. 2	Exp. 1	Exp. 2
ΔEV	0.65	0.85	0.09	0.1	58.09	85.31	<0.001	<0.001	0.53	0.66
Δuncertainty	0.38	0.32	0.06	0.07	35.32	23.59	<0.001	<0.001	0.42	0.35
Age	0.12	-0.09	0.08	0.07	2.62	1.89	0.106	0.17	0.11	0.10
Instructions comprehension	0.01	0.03	0.08	0.07	0.04	0.21	0.85	0.645	0.01	0.03
EV comprehension	-0.1	0.14	0.08	0.07	2.3	5.15	0.129	0.023	0.11	0.16
Correct fishing	-0.01	0	0.08	0.07	0.01	0	0.923	0.945	0.01	0
ΔEV * Age	0.14	0.14	0.09	0.09	2.83	2.61	0.092	0.106	0.12	0.12
Δuncertainty * Age	0.24	0.15	0.06	0.06	15.42	6.06	<0.001	0.014	0.27	0.18

Supplementary Table 3. The effects of EV and uncertainty are observed even in the absence of instrumental utility. Model results from a logistic mixed-effects regression predicting information-seeking in children in Experiments 1 and 2. The model was similar to the one described in the main text, but the analyses were run only on trials were Δagency was zero; thus, Δagency was not included as a predictor. Therefore, the model had random intercepts for each participant, fixed effects and random slopes for ΔEV and Δuncertainty, and fixed effects for age and its interaction with each included Δ, for instructions comprehension scores, EV comprehension scores. As in the main analyses, we found significant effects of ΔEV and Δuncertainty, even though instrumental utility was constant across both sides of the pond. Importantly, the interaction between age and Δuncertainty remained significant.

Supplementary Methods

Information-seeking task instructions

In the study, each of the below images was shown one at a time. Participants were free to navigate the instructions at their own pace. The text reported below was both shown and read out loud through a recording. The following modifications were made for Experiment 3 and 4. Experiment 3: all references to hidden items (images 40-44) were removed. Experiments 4: an alternative type of seaweed, which did not hide any item, was introduced between page 44 and 45 (see images 1s-4s). Fisherman icon: Flaticon.com. Garbage truck: Freepik.com

1 Welcome to the experiment. You are about to hear the story behind our game, so listen closely!
You can click the button below, which says “Next”.

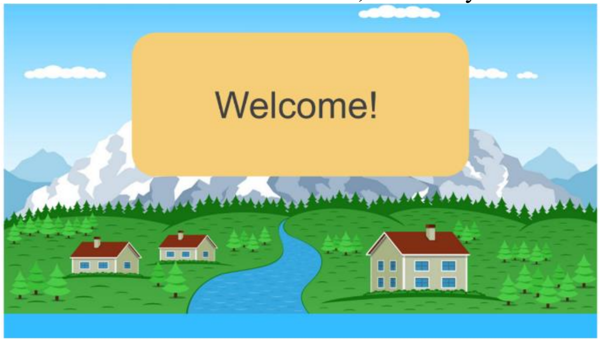

2 Once upon a time, in a nice little village on a river, lived two fishermen who were identical twins. They were known as the 'Fishertwins'.

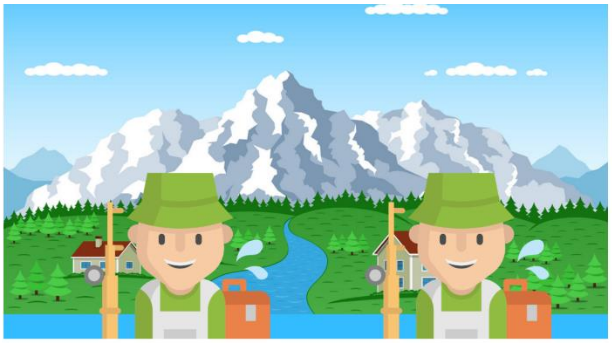

3 Sometimes, they would get some VERY BIG fish. Other times, they would only get smaller ones.

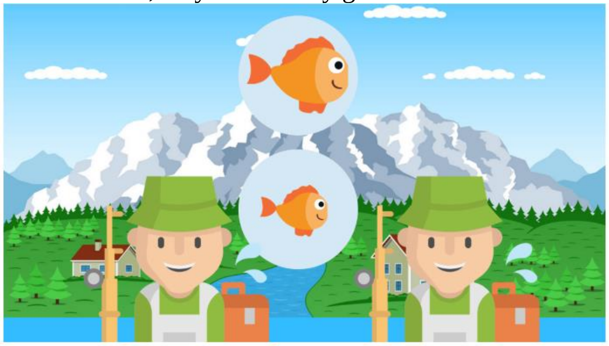

4 But one day, a garbage truck came too close to the river…

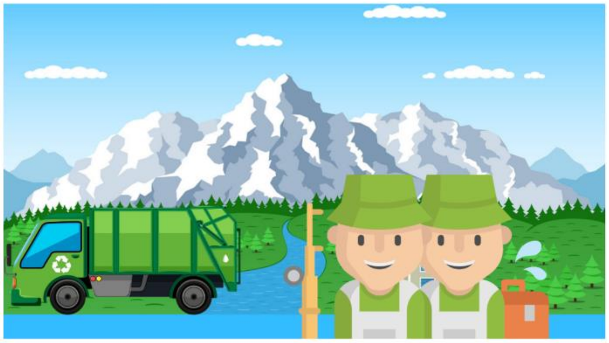

5 ... Accidentally opened its back door...

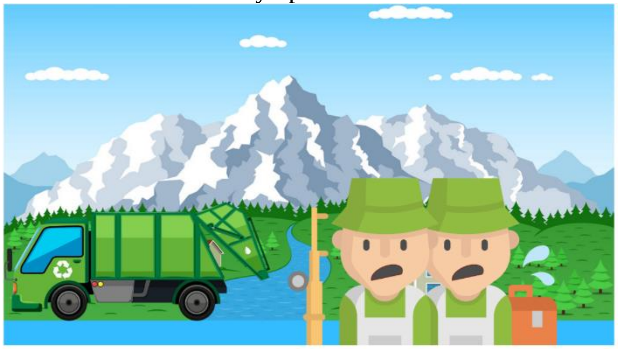

6 ... And let all the garbage fall into the river!

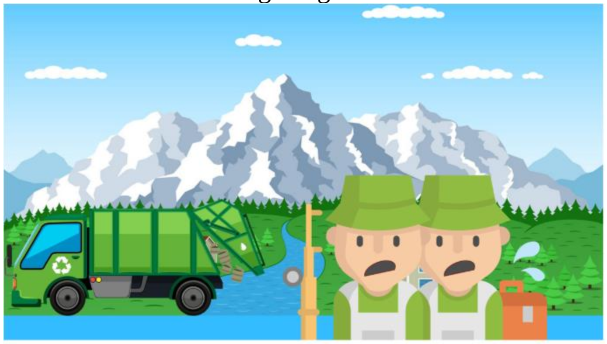

7 Oh no! Now the Fishertwins are all covered in grease and the river is full of trash!

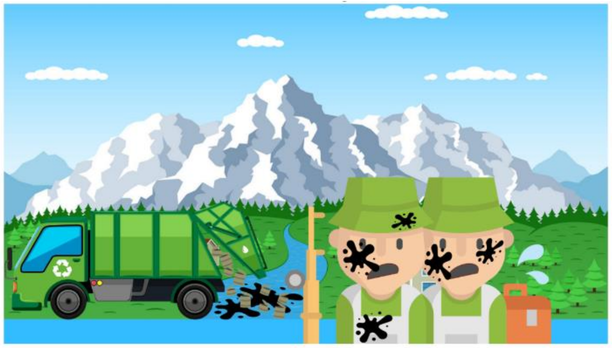

8 The Fishertwins still have some dirt in their eyes and can't see well.

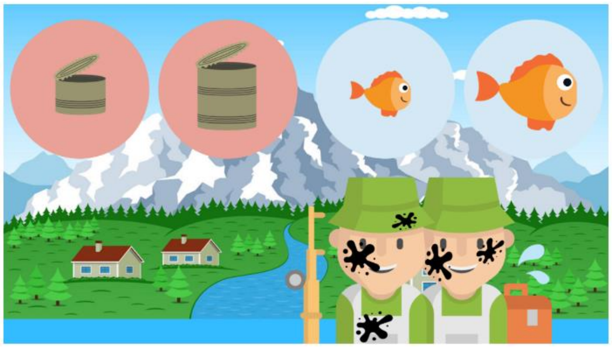

9 They are going to need your help to make sure they fish as many fish as possible, and avoid putting cans into their fishing buckets: fishing empty cans and putting them in the bucket will ruin the fish that is already there.

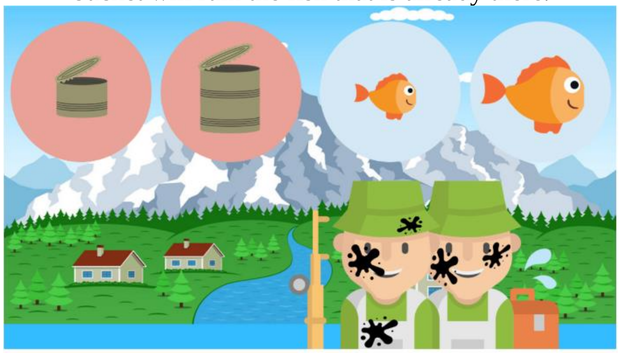

10 In this game, big fish are worth twice as much as small fish. Big cans are twice as bad as small cans.

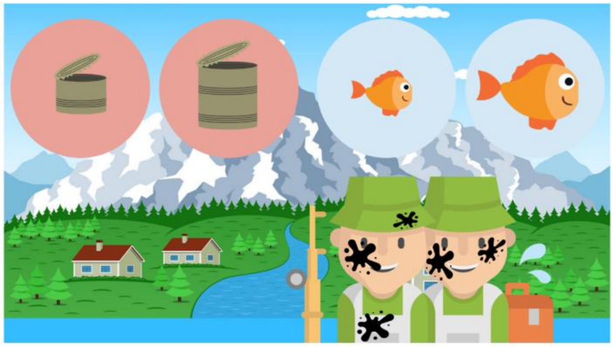

11 The two Fishertwins are fishing on opposite sides of the river.

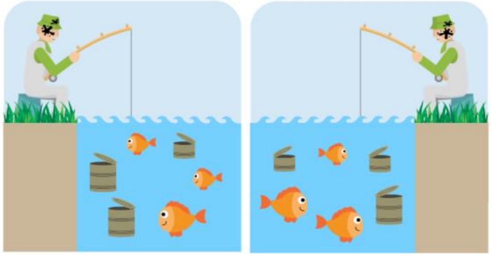

12 One of the items you see in the river is already attached to the hook but you can’t see the hook so you don’t know if it is a big fish, a small fish, a big can or a small can.

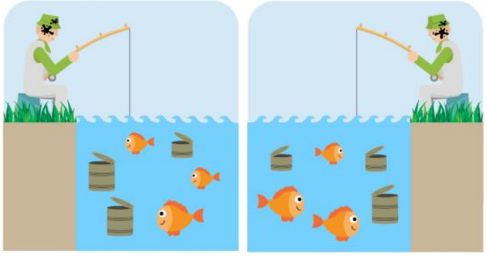

13 In fact, it does not matter where the fishing pole is: the hook under the water could look like this.

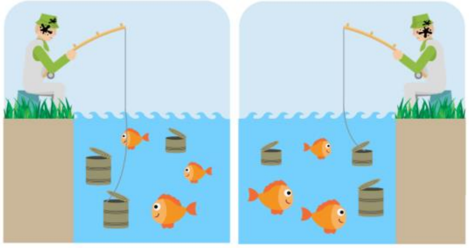

14 Or maybe like this. You just don't know.

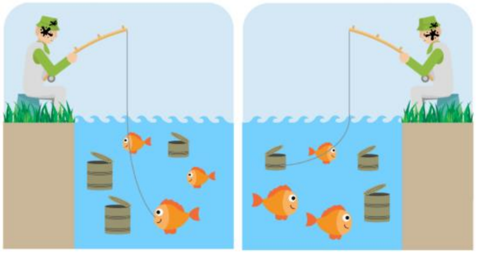

15 Some of the fish and cans are covered in grease from the trash, which made them super slippery.

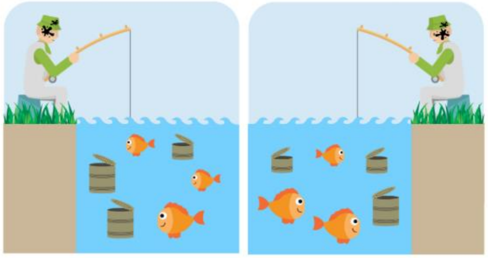

16 So even if you see them in the river, they cannot get attached to the hook...

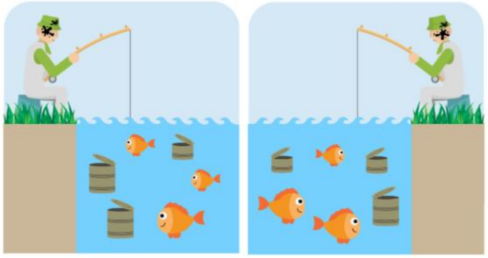

17 Knowing which ones are covered in grease can help you decide whether it's a good idea to lift the fishing pole or if it's better not to risk it for that time...

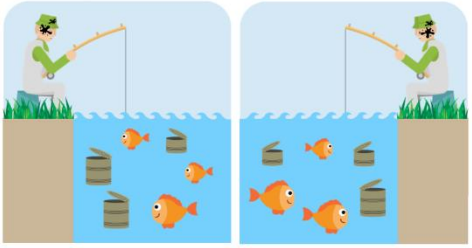

18 Luckily, you have been given a special flashlight that can show you only the fish and cans that are clean enough to be caught, and hide away the ones that are too greasy...

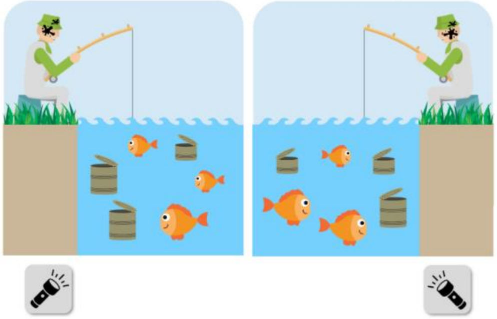

19 There's only one problem: you can only use the flashlight on one side of the river at a time.

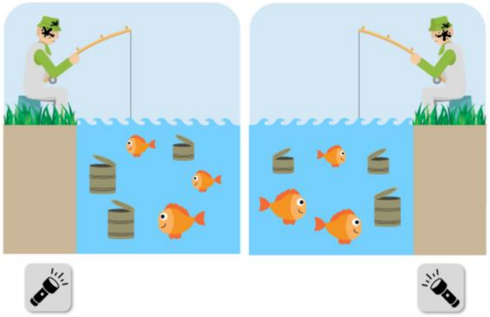

20 In the game, you should click on one of the flashlight buttons to use the flashlight on that side.
You will get to pick where to use this special tool each time.

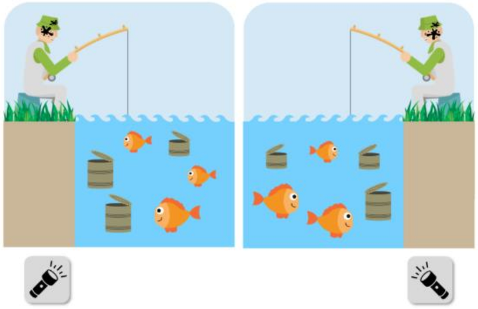

21 Look at what happens if I click here.

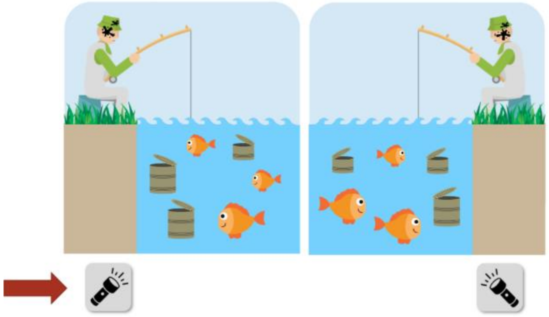

22 Now only the non-greasy objects are seen!

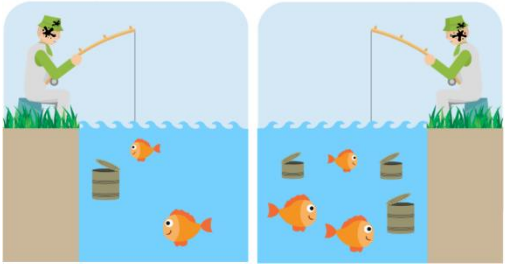

23 Because the Fishertwins can’t see well, they cannot see what happens when you use the flashlight
- only you can see which objects are not greasy.

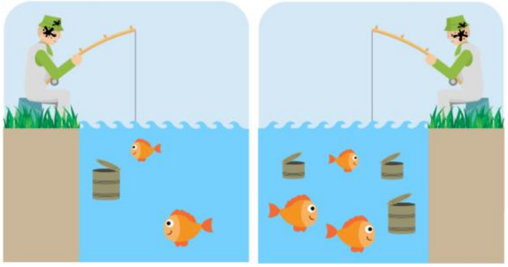

24 Each time, the Fishertwins will have to decide whether they want to lift the fishing pole and put whatever was attached to it in their bucket, or wait till the situation becomes better.

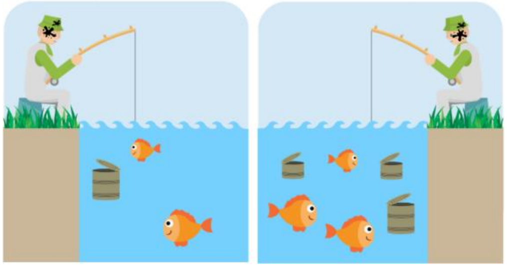

25 If they always decide to fish, they will likely get plenty of fish but also plenty of empty cans, and that will ruin all the fish.

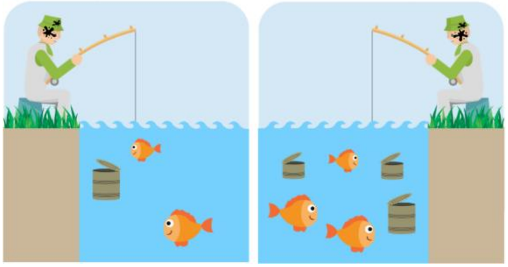

26 But if they never try, they will never get good fish to bring home to their family. So, it is important to pick wisely each time according to what is in the water!

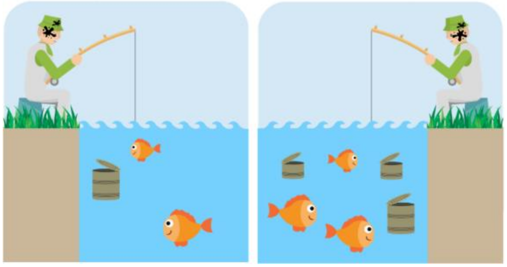

27 The Fishertwins cannot see well because of the grease and dirt, so sometimes they will ask you to decide whether they should lift their fishing pole or not.

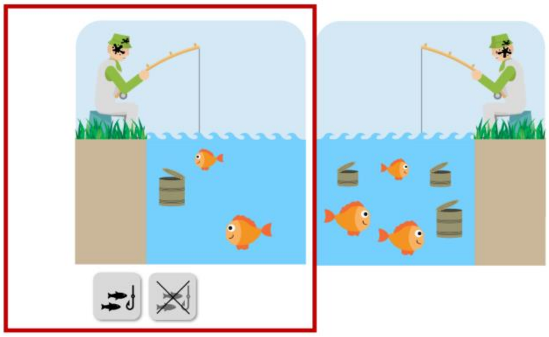

28 Look at the pictures below.

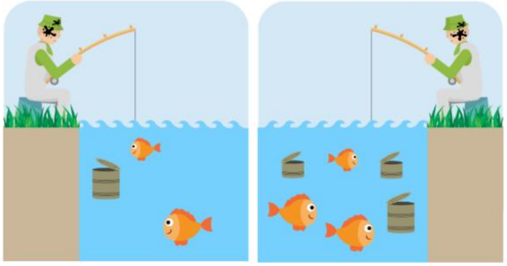

29 This is you!

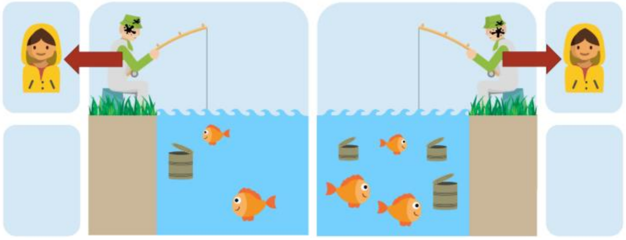

30 When only your picture is seen, it means the Fishertwin will ask you to decide whether to lift the pole or not.

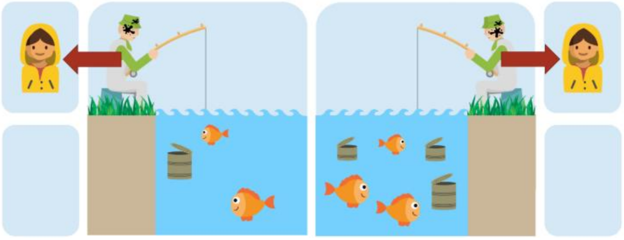

31 When only the Fishertwin's picture is seen, it means he will decide himself whether to lift the pole or not.

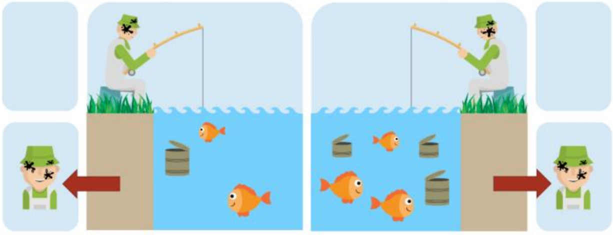

32 When you see both the Fishertwin's picture and your own, it means sometimes he will ask you to decide whether to lift the pole and sometimes he will decide himself.

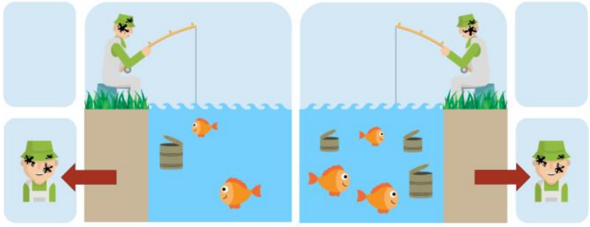

33 Then, if one or both Fishertwins ask you to decide whether they should lift the fishing pole and put their catch in the bucket...

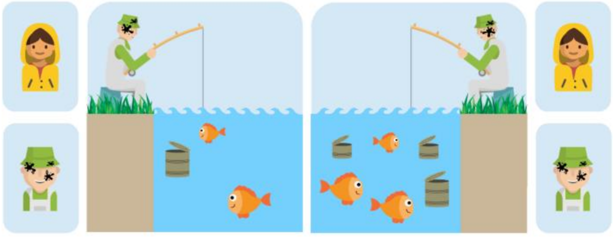

34 ... you will decide if you want to fish or not.

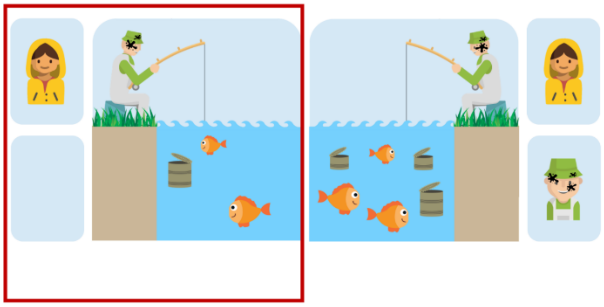

35 In the game, you should click this button if you want to fish…

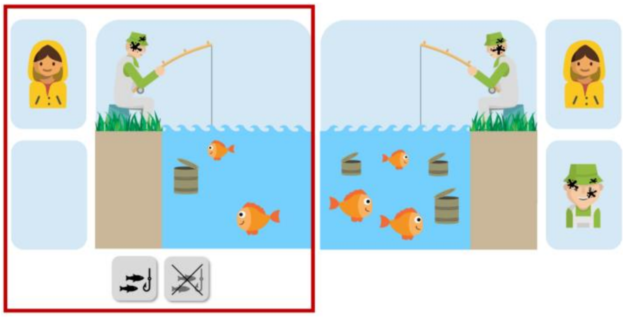

36 Or this button if you DON'T want to fish.

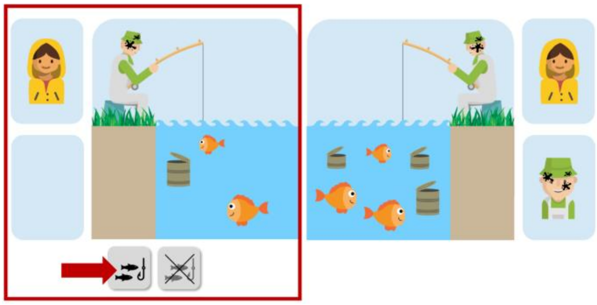

37 Otherwise, you will just wait while the fisherman is choosing.

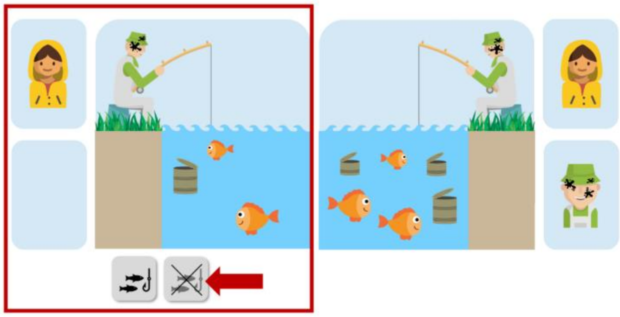

38 Note that what happens to one of the Fishertwins is separate from what happens to the other one.

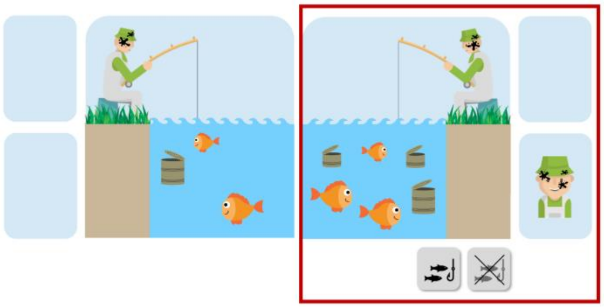

39 Note that what happens to one of the Fishertwins is separate from what happens to the other one.

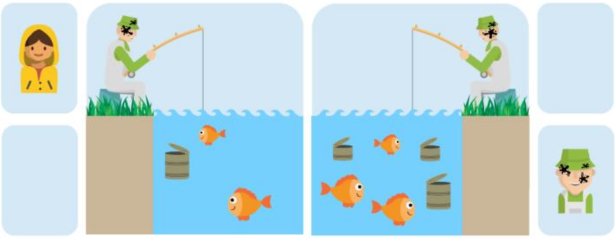

40 Sometimes, some seaweed might hide what is in the water.

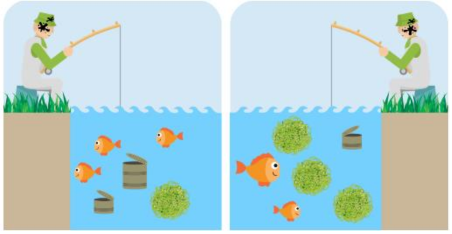

41 Luckily, the flashlight also lets you see through the seaweed.

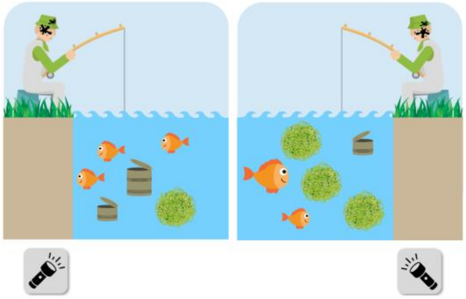

42 But only on the side where you click! For example, if I click here...

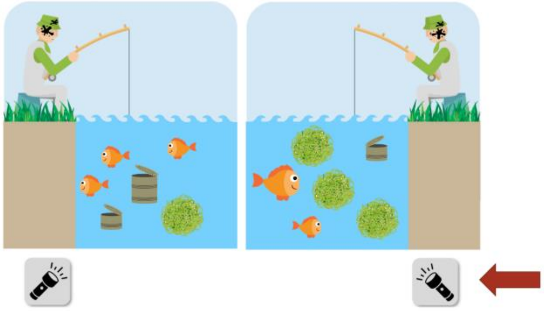

43 ... the seaweed will be gone...

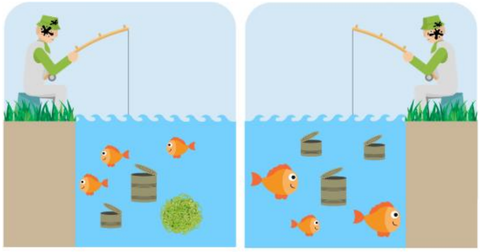

44 ...and you’ll only see the non-greasy items for the side you chose.

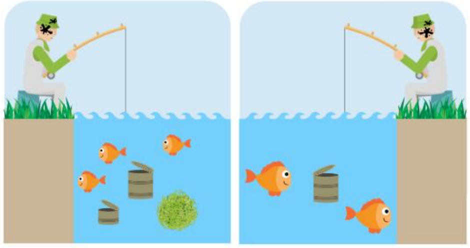

45 Finally, sometimes we'll ask you a different question just to check that you are paying attention.

46 For example, we could ask you if you see only fish...

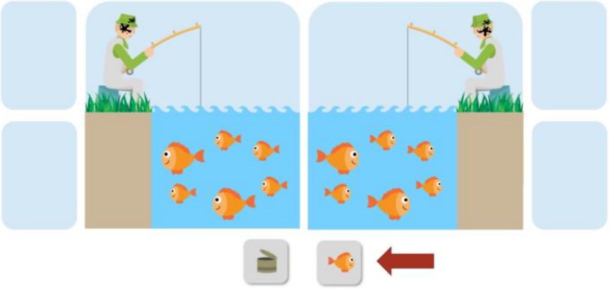

47 ...or only cans.

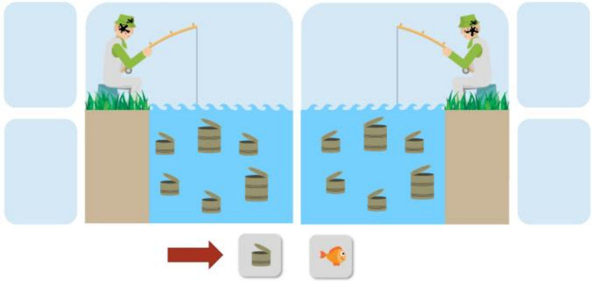

48 I know this is a lot of information for you to remember, so I'm going to ask you a couple of questions to make sure everything is clear.

49 You won't be able to know how well you are doing till the very end, so keep trying your best!

50 Click "Next" when you are ready!

Additions for Experiment 2 (between pages 44 and 45):

1s But there is also a different kind of seaweed. This type of seaweed has holes in it, so you know that there is nothing behind it.

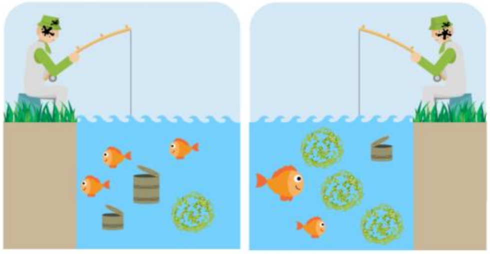

2s So, in the game, when you click on the flashlight...

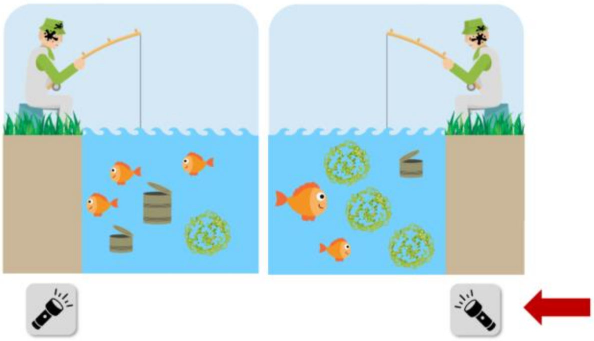

3s ...the seaweed will be gone...

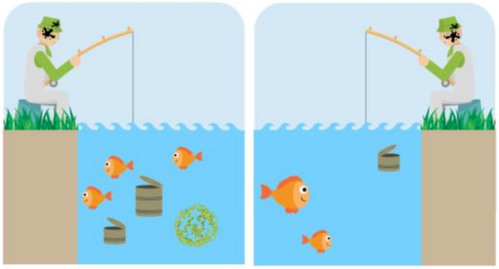

4s ...and you'll only see the non-greasy items for the side you chose.

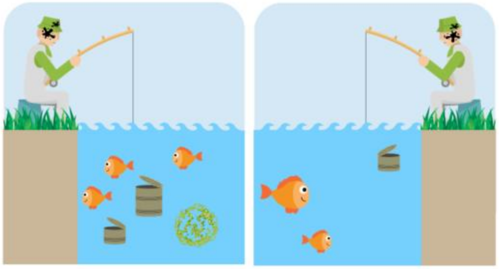

Instructions comprehension task

Participants answered one question at a time by selecting one of the buttons displayed above each option. If they answered correctly, they were presented with the next one. If not, they were prompted to try and answer the question again. Participants could attempt each question three times at most to avoid being excluded from the dataset. The text shown below was both presented to participants and read aloud through a recording. Below, square brackets indicate the correct answer. The following modifications were implemented for Experiments 3 and 4. Experiment 3: Questions 12-13 were removed. Experiment 4: Text for questions 12 and 13 was slightly changed into "[…] this *type* of seaweed […]" in place of "[…] the seaweed […]". Two additional questions were added (see questions 1s and 2s). Fisherman icon: Flaticon.com.

1 Which one is better? Big fish or big can? [Big fish]

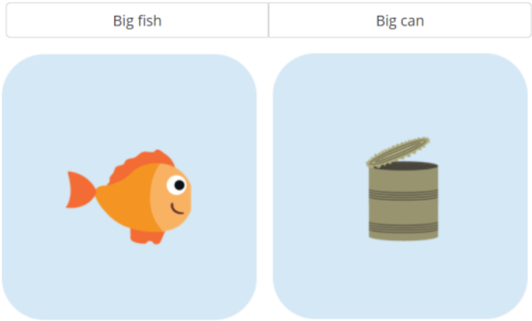

2 Which one is better? Big fish or small fish? [Big fish]

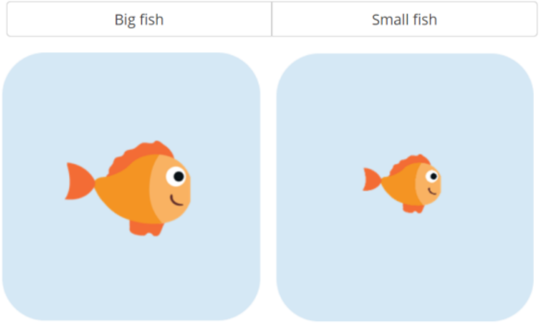

3 Which one is better? Small fish or small can? [Small fish]

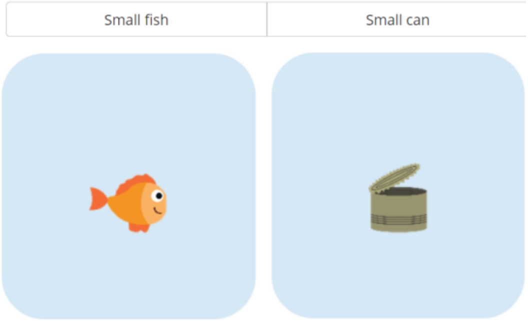

4 Which one is worse? Big can or small can? [Big can]

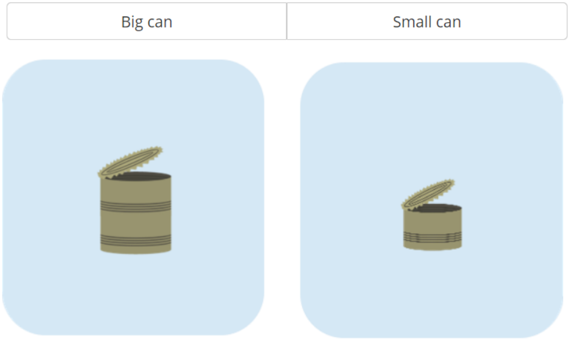

5 Who will decide whether to fish or not when you see this? You, the Fishertwin, or we don't know yet? [You]

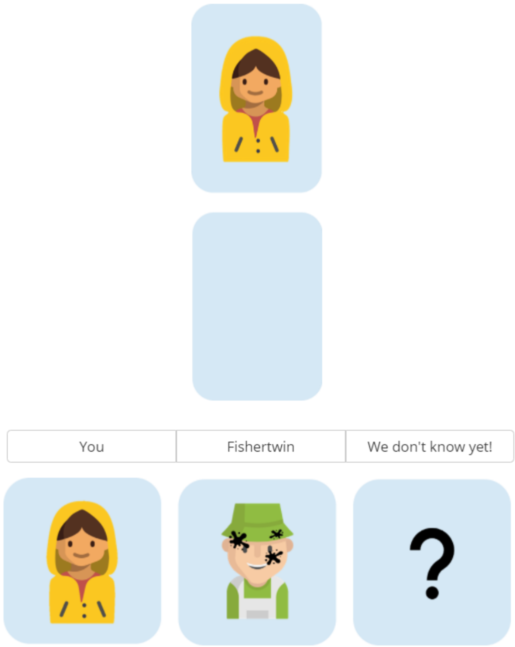

6 Who will decide whether to fish or not when you see this? You, the Fishertwin, or we don't know yet? [The Fishertwin]

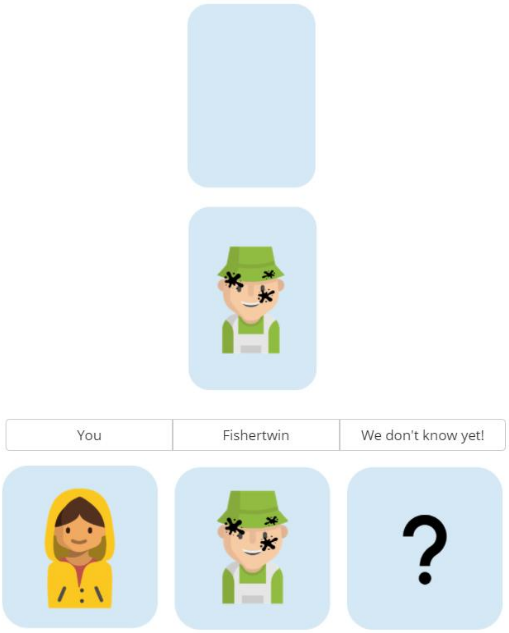

7 Who will decide whether to fish or not when you see this? You, the Fishertwin, or we don't know yet? [We don't know yet]

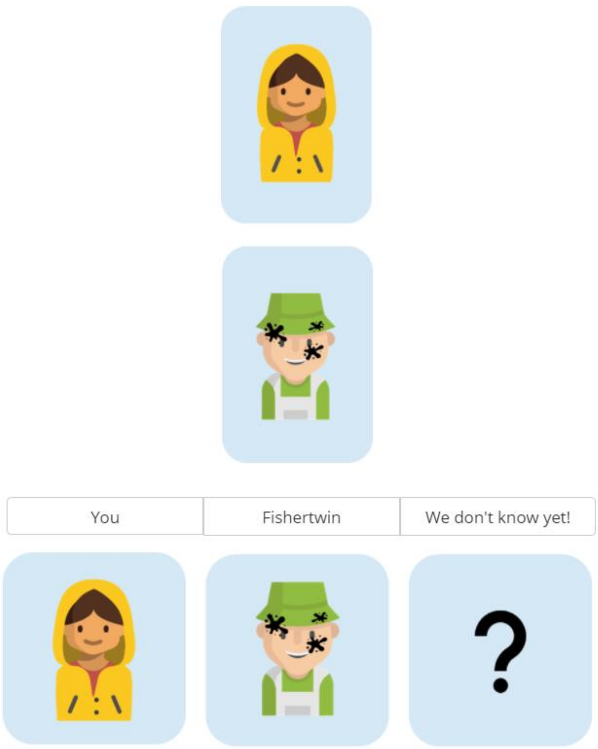

8 What happens if you click on the flashlight button? No objects will be shown, only the non-greasy objects will be shown, or nothing happens? [Only the non-greasy objects will be shown]

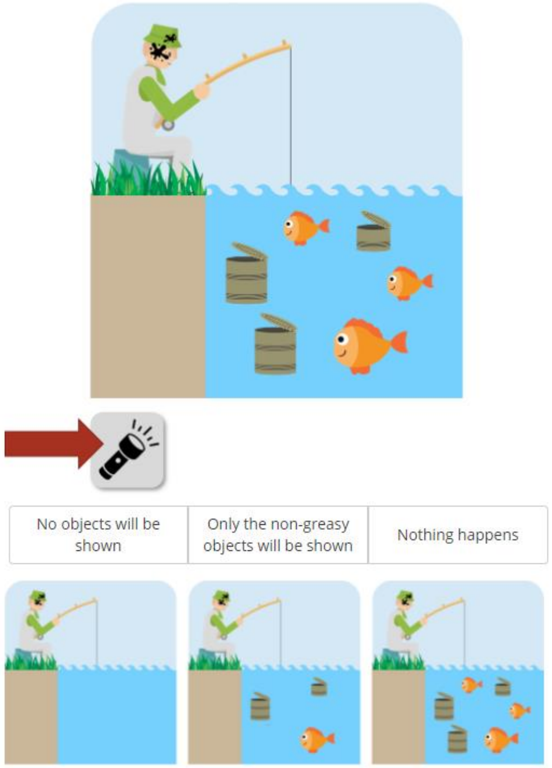

9 What happens if you click this button? You will fish and the Fishertwin will put your catch in the basket, or you will not fish? [You will fish and the Fishertwin will put your catch in the basket]

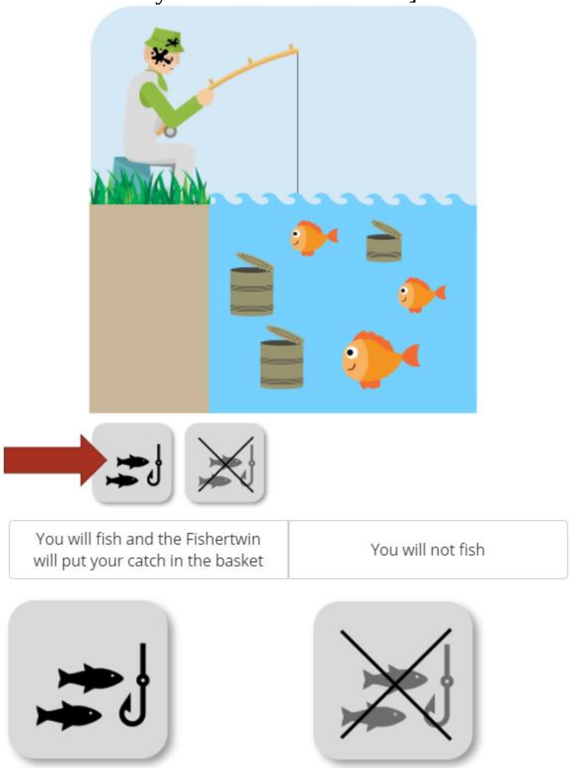

10 What happens if you click this button? You will fish and the Fishertwin will put your catch in the basket, or you will not fish? [You will not fish]

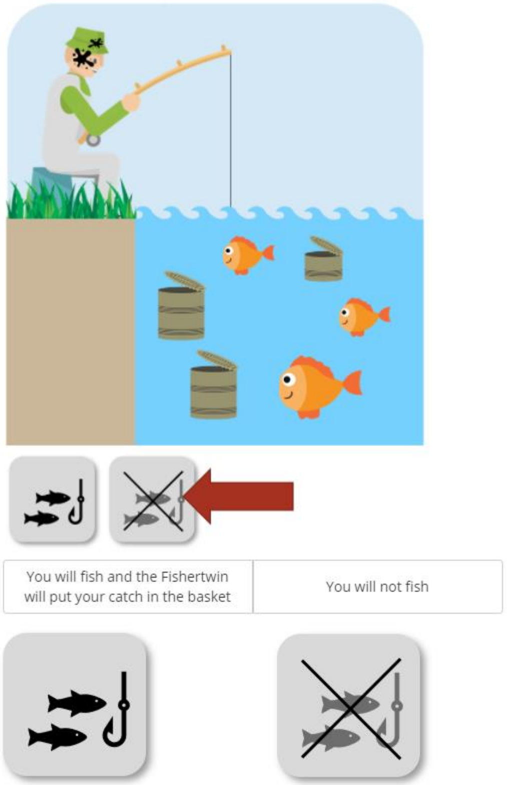

11 Can the Fishertwin see what happens when you use the flashlight? Yes or no? [No]

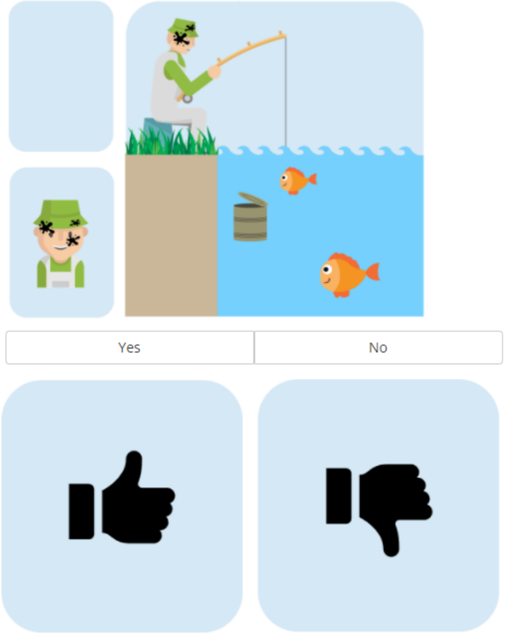

12 Is there something behind the seaweed? Yes, no, or maybe? [Yes or Maybe]

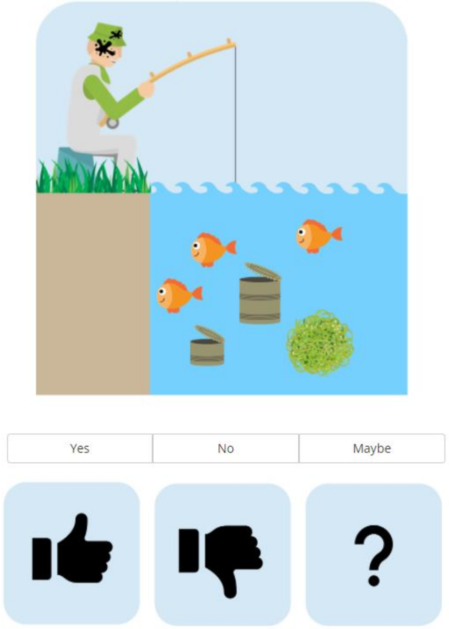

13 What's behind the seaweed? A fish, a can, or we don't know? [We don't know]

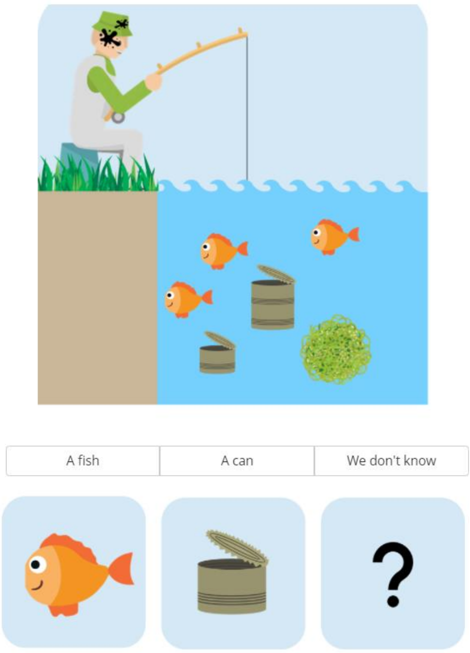

Additions for Experiment 4 (after question 13):

1s Is there something behind this type of seaweed? Yes, no, or maybe? [No]

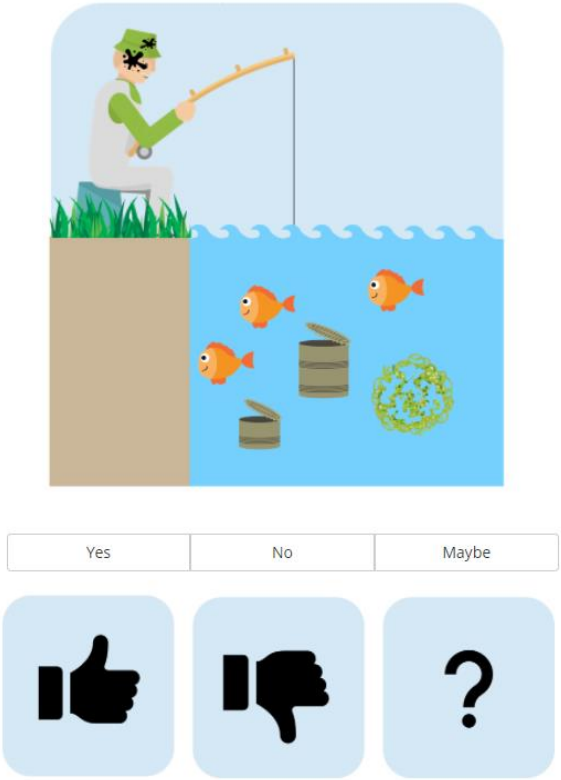

2s What's behind the seaweed? A fish, a can, or nothing? [Nothing]

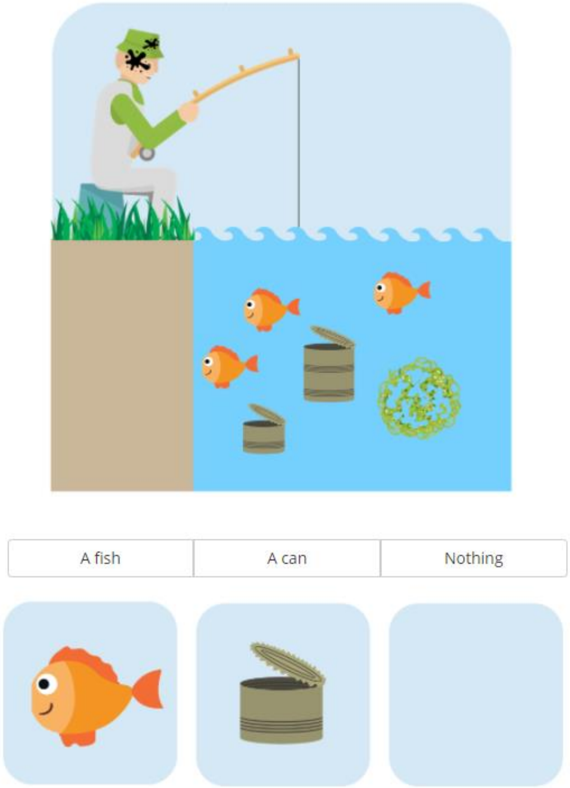

Expected values comparison task instructions

In the study, each of the below images was shown one at a time. Participants were free to navigate the instructions at their own pace. The text reported below was both shown and read out loud through a recording. For Experiments 3 and 4, only pages 1-3 were presented, as participants did not have to complete the expected values task. Fisherman icon: Flaticon.com.

1

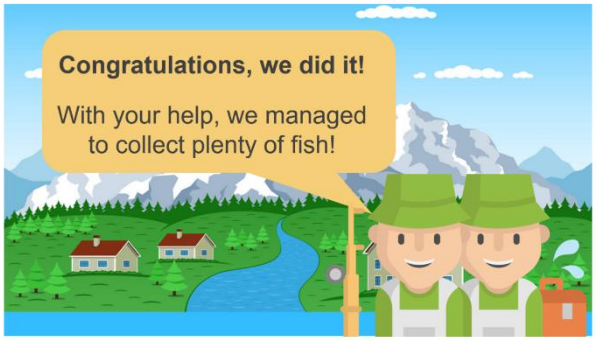

2

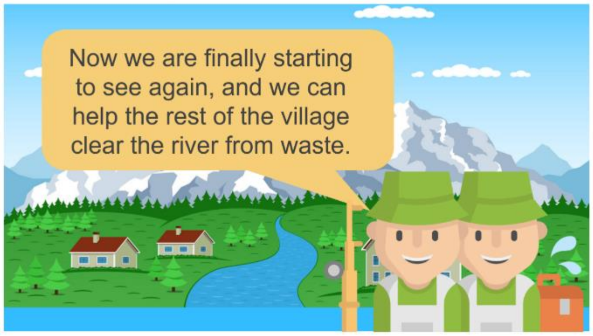

3

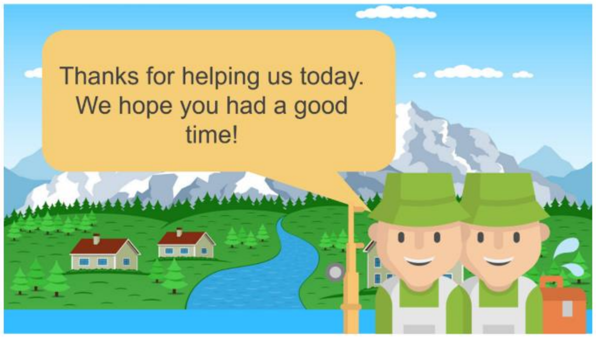

4

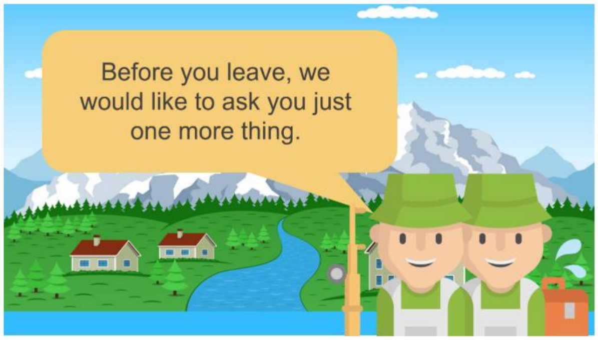

5

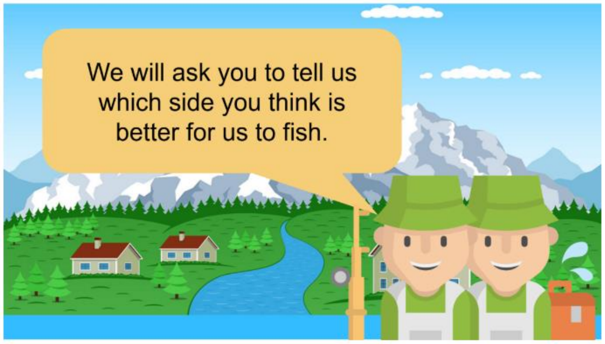

6 Once the game starts, you should click on the arrow for the side you think is better.

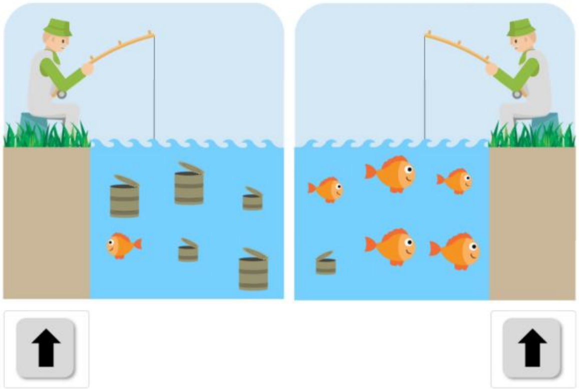

7 In the game, if you think the left side is better, you should click on the button that you will see here.

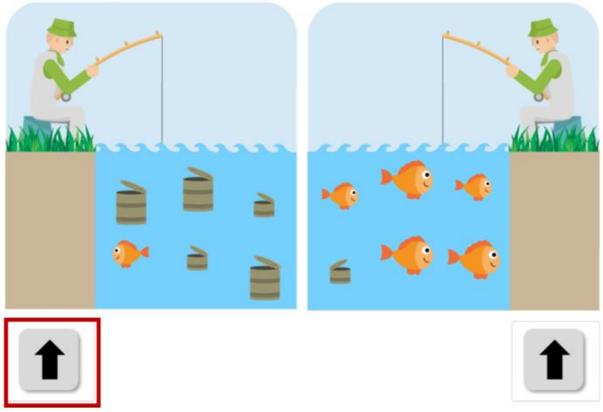

8 If you think the right side is better, you should click on a button that looks like this.

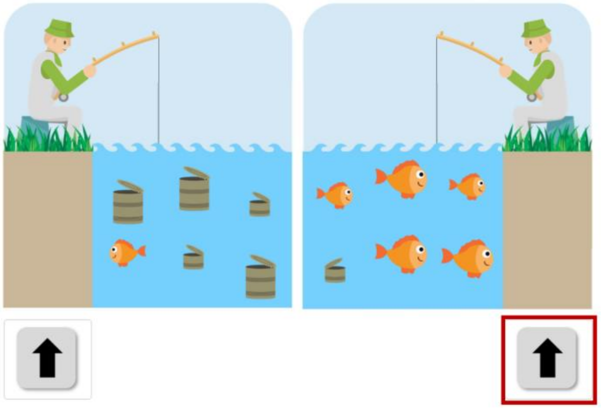

9

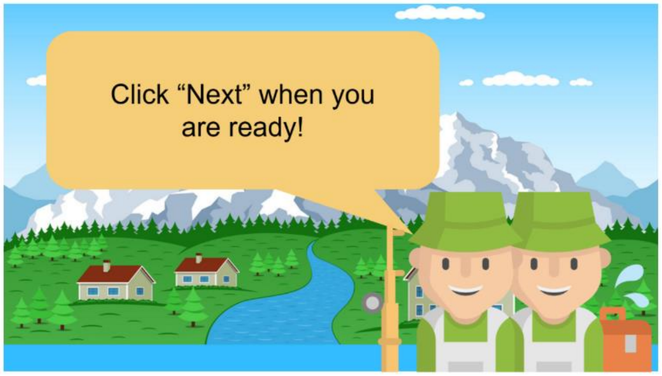

<u>Uncertainty tracking task (Experiment 5) instructions</u>

In the study, each of the below images was shown one at a time. Participants were free to navigate the instructions at their own pace. The text reported below was both shown and read out loud through a recording. Fisherman icon: Flaticon.com.

1 Please listen to the instructions before pressing any button. Let's begin!

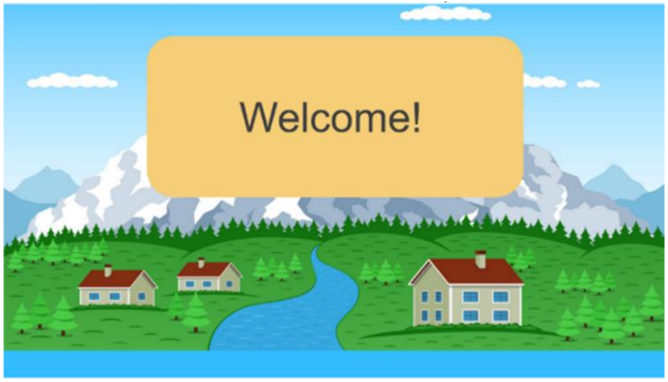

2 Two fishermen live in a little village with a big river. The river has a lot of fish but also a lot of cans. Sometimes the fishermen will catch a fish. Other times they will catch a can.

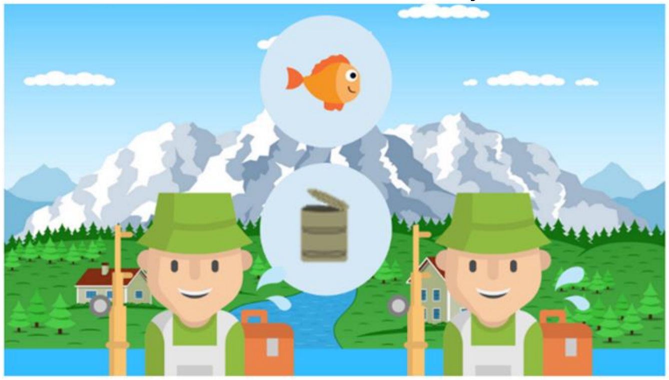

3 The fishermen like to fish on opposite sides of the river and they can only catch one of the items on the side of the river in front of them. But the fishermen cannot control where exactly the hook will go. So sometimes they may catch a near item and sometimes a far item.

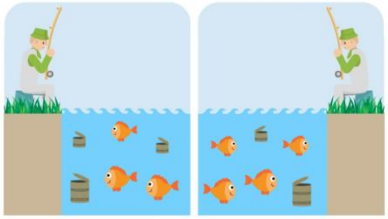

4 Sometimes there is also seaweed in the river. Either fish or cans hide behind the seaweed. We don't actually know what is behind the seaweed. Sometimes the fishermen will catch what is hidden behind the seaweed. It may be a fish or a can.

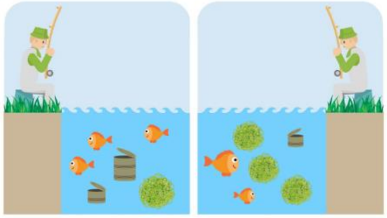

5 Your task is to guess as best as you can what each fisherman is going to catch from the river in front of them.

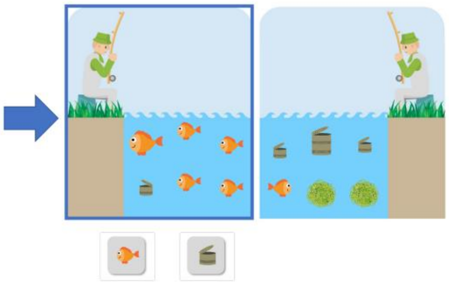

6 For example, we might ask you on the left side first: What is this fisherman going to catch? If you think that the fisherman is going to catch a fish you should click the fish button. If you think that the fisherman is going to catch a can you should click the can button. Don't forget that the fisherman may also catch a fish or a can hidden behind the seaweed.

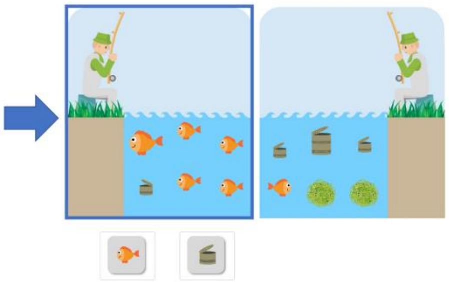

7 Sometimes you may be very sure that the fisherman will catch a fish or a can, sometimes not at all sure.
After you have guessed we will ask you 'How sure are you?'

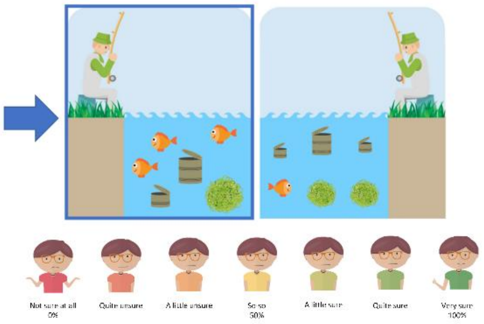

8 If you are very sure whether the fisherman will catch a fish or a can, you should click on the person with their thumbs up.

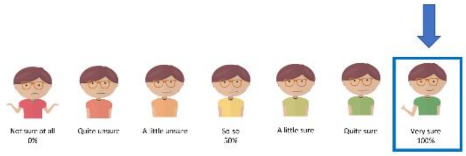

9 If you are not sure at all, you should click on the person with their hands wide open.

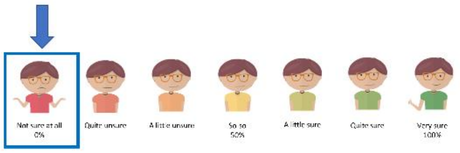

10 The surer you are, the closer you should click to the person with their thumbs up. So for example if you are quite sure but not very sure, you should click on one of the icons below:

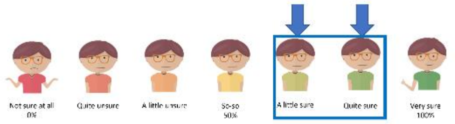

11 If you are so-so sure click on the middle person with the yellow shirt:

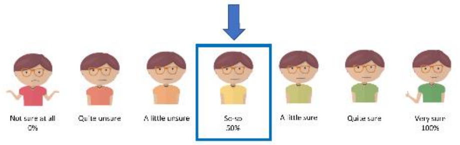

12 If you are just a tiny bit sure click on the person next to the one with the red shirt. And if you are a little bit surer click on the next one over.

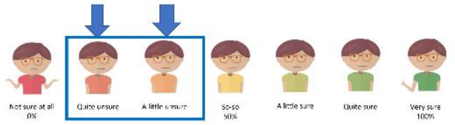

13 I know this is a lot of information. Let's practice.

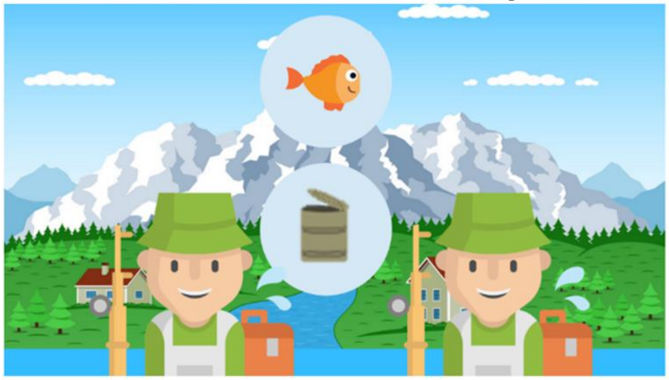

<u>Instructions comprehension task for the uncertainty tracking task (Experiment 5)</u>

Participants answered one question at a time by selecting one of the buttons displayed above each option. If they answered correctly, they were presented with the next one. If not, they were prompted to try and answer the question again. Participants could attempt each question three times at most to avoid being excluded from the dataset. The text shown below was both presented to participants and read aloud through a recording. Below, square brackets indicate the correct answer.

1 What are you going to click if you think that the fisherman will catch a can? [Can]

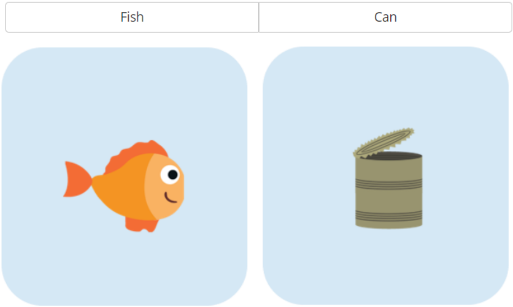

2 What are you going to click if you think that the fisherman will catch a fish? [Fish]

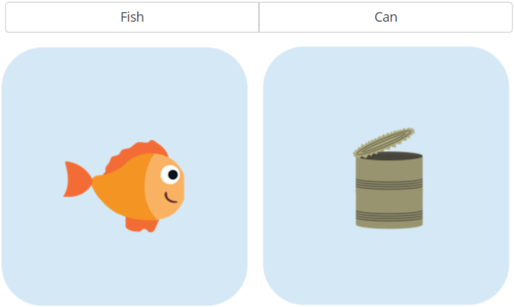

3 What are you going to click if you are very sure? [Very sure 100%]

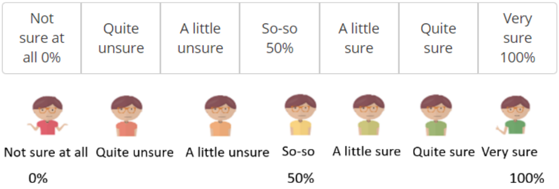

4 What are you going to click if you are very sure? [Not sure at all 0%]

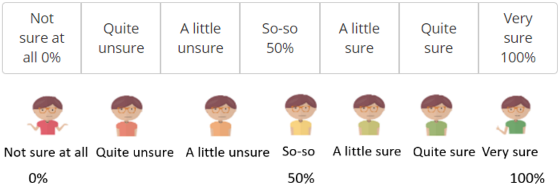

5 What's behind the seaweed? A fish, a can, or we don't know? [We don't know]

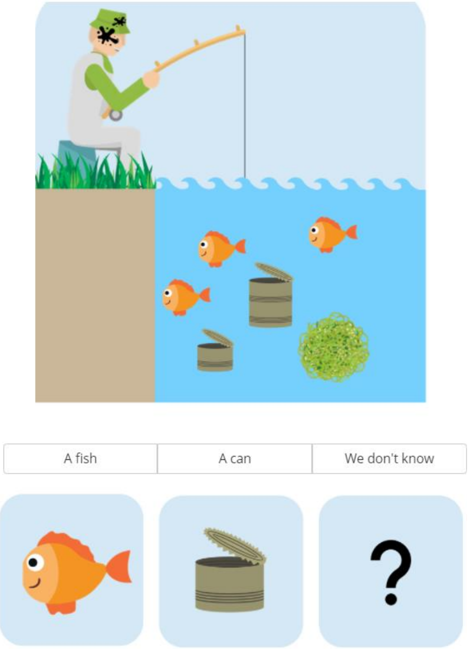

6
