# Supplementary material for: Multifaceted information-seeking motives in children
Source: Nat Commun. 2023 Sep 7;14:5505. doi: 10.1038/s41467-023-40971-x (PMC10485006; doi:10.1038/s41467-023-40971-x)
Supplement: Supplementary file 2 — Reporting Summary [file 41467_2023_40971_MOESM2_ESM.pdf]

nature portfolio

Corresponding author(s): Gaia Molinaro, Tali Sharot

Last updated by author(s): Aug 7, 2023

Reporting Summary

Nature Portfolio wishes to improve the reproducibility of the work that we publish. This form provides structure for consistency and transparency in reporting. For further information on Nature Portfolio policies, see our Editorial Policies and the Editorial Policy Checklist.

Statistics

For all statistical analyses, confirm that the following items are present in the figure legend, table legend, main text, or Methods section.

n/a	Confirmed	
☐	☒	The exact sample size (*n*) for each experimental group/condition, given as a discrete number and unit of measurement
☐	☒	A statement on whether measurements were taken from distinct samples or whether the same sample was measured repeatedly
☐	☒	The statistical test(s) used AND whether they are one- or two-sided<br>*Only common tests should be described solely by name; describe more complex techniques in the Methods section.*
☐	☒	A description of all covariates tested
☒	☐	A description of any assumptions or corrections, such as tests of normality and adjustment for multiple comparisons
☐	☒	A full description of the statistical parameters including central tendency (e.g. means) or other basic estimates (e.g. regression coefficient) AND variation (e.g. standard deviation) or associated estimates of uncertainty (e.g. confidence intervals)
☐	☒	For null hypothesis testing, the test statistic (e.g. *F*, *t*, *r*) with confidence intervals, effect sizes, degrees of freedom and *P* value noted<br>*Give P values as exact values whenever suitable.*
☒	☐	For Bayesian analysis, information on the choice of priors and Markov chain Monte Carlo settings
☐	☒	For hierarchical and complex designs, identification of the appropriate level for tests and full reporting of outcomes
☐	☒	Estimates of effect sizes (e.g. Cohen's *d*, Pearson's *r*), indicating how they were calculated

Our web collection on statistics for biologists contains articles on many of the points above.

Software and code

Policy information about availability of computer code

Data collection	The experiments were designed using Gorilla Experiment Builder. Participants were recruited using Lookit (children) and Prolific (adults; version numbers NA).
Data analysis	Analyses were conducted in R (version 4.2.0). Used packages include: glmnet (version 4.1-4), lme4 (version 1.1-29), interactions (version 1.2.0), modelbased (version 0.8.5), and BayesFactor (version 0.9.12-4.3). Custom code has been made available at https://github.com/affective-brain-lab/information-seeking-children/.

For manuscripts utilizing custom algorithms or software that are central to the research but not yet described in published literature, software must be made available to editors and reviewers. We strongly encourage code deposition in a community repository (e.g. GitHub). See the Nature Portfolio guidelines for submitting code & software for further information.

Data

Policy information about availability of data

All manuscripts must include a data availability statement. This statement should provide the following information, where applicable:

- Accession codes, unique identifiers, or web links for publicly available datasets
- A description of any restrictions on data availability
- For clinical datasets or third party data, please ensure that the statement adheres to our policy

All data have been made publicly available at a dedicated GitHub repository (https://github.com/affective-brain-lab/information-seeking-children/), which has also been released on Zenodo (https://zenodo.org/record/8151641). Raw data has been anonymized and consolidated into a smaller number of files to increase

readability; information in html strings regarding the stimuli presented was transformed into measures of interest (EV, uncertainty, etc.).

Research involving human participants, their data, or biological material

Policy information about studies with human participants or human data. See also policy information about sex, gender (identity/presentation), and sexual orientation and race, ethnicity and racism.

Reporting on sex and gender	Sex was based on self- or parent-reported information.
Reporting on race, ethnicity, or other socially relevant groupings	NA
Population characteristics	For children, age and gender characteristics were as follows:<br>Experiment 1: N = 204, age M =7.44 ± 2.38, age range = 4-12 years, 47.5% female.<br>Experiment 2: N = 196, age M = 7.62 ± 2.27, age range = 4-11 years, 48% female.<br>Experiment 3: N = 27, age M = 6.93 years ± 2.6 years, age range = 4-12 years, 59.3% female.<br>Experiment 4: N = 40, age: M = 7.45 years ± 2.57 years, age range = 4-12 years, 42.5% female.<br>Experiment 5: N = 54, age: M = 7.24 years ± 2.49 years, age range = 4-12 years, 51.85% female.<br>For adults, age and gender characteristics were as follows:<br>Experiment 6: N= 28, age M = 28.6 ± 11.50 years, age range = 18-66 years, 32.1% female<br>Experiment 7: N = 29, age M = 40.2 ± 11.70, age range = 22-64 years, 34.5 % female).
Recruitment	All data was collected via Lookit (children) and Prolific (adults). Access to these platforms requires Internet connection and access to a desktop or laptop computer. These factors may have biased participant recruitment. We are not aware of any other selection biases.
Ethics oversight	The Massachusetts Institute of Technology Committee on the Use of Humans as Experimental Subjects provided ethical approval for recruitment of children in this study. The University College London Research Ethics Committee approved the recruitment of adult participants for this study.

Note that full information on the approval of the study protocol must also be provided in the manuscript.

Field-specific reporting

Please select the one below that is the best fit for your research. If you are not sure, read the appropriate sections before making your selection.

☐ Life sciences ☒ Behavioural & social sciences ☐ Ecological, evolutionary & environmental sciences

For a reference copy of the document with all sections, see nature.com/documents/nr-reporting-summary-flat.pdf

Behavioural & social sciences study design

All studies must disclose on these points even when the disclosure is negative.

Study description	This study investigated different motives of information-seeking throughout development. All experiments were quantitative cross-sectional studies.
Research sample	For children, age and gender characteristics were as follows:<br>Experiment 1: N = 204, age M =7.44 ± 2.38, age range = 4-12 years, 47.5% female.<br>Experiment 2: N = 196, age M = 7.62 ± 2.27, age range = 4-11 years, 48% female.<br>Experiment 3: N = 27, age M = 6.93 years ± 2.6 years, age range = 4-12 years, 59.3% female.<br>Experiment 4: N = 40, age: M = 7.45 years ± 2.57 years, age range = 4-12 years, 42.5% female.<br>Experiment 5: N = 54, age: M = 7.24 years ± 2.49 years, age range = 4-12 years, 51.85% female.<br>For adults, age and gender characteristics were as follows:<br>Experiment 6: N= 28, age M = 28.6 ± 11.50 years, age range = 18-66 years, 32.1% female<br>Experiment 7: N = 29, age M = 40.2 ± 11.70, age range = 22-64 years, 34.5 % female).<br>For Experiments 1, 2, 6, and 7, N was determined based on a power analysis conducted on pilot data to achieve power of 80% with alpha = 0.05 using the G*Power 3 software. For Experiments 3, 4, and 5, N was based on achieved power in Experiments 1 and 2.
Sampling strategy	All experiments implemented a convenient sampling strategy (online participants).<br>Initial sample sizes were calculated based on a pilot study. Power analysis was conducted using G*power (http://www.psychologie.hhu.de/arbeitsgruppen/allgemeinepsychologieund-arbeitspsychologie/gpower.html), with .80 power and α = 0.05.
Data collection	All data was collected online using Lookit (children) and Prolific (adults). Participants completed the experiment at their own pace without the experimenter's supervision. Parents may have assisted children with online navigation, but were asked not to interfere with the children’s decisions.

Timing	Data was collected between 01/27/2021 and 12/12/2022.
Data exclusions	Experiment 1: 4 children were excluded due to failure in one of the attention checks. Data from 1 child were lost due to a technical issue with the experiment server.<br>Experiment 2: 4 children were excluded due to failure in one of the attention checks.<br>Experiment 3: 2 children were excluded due to failure in one of the attention checks.<br>Experiment 4: 1 child was excluded due to failure in one of the attention checks.<br>Experiment 5: 1 child was excluded due to failure in one of the attention checks.<br>Experiment 6: 1 adult was excluded due to failure in one of the attention checks.<br>Experiment 7: no participants were excluded.
Non-participation	No participants declined participation.
Randomization	The experiments focused on within-subjects effects and no randomization into groups was required.

Reporting for specific materials, systems and methods

We require information from authors about some types of materials, experimental systems and methods used in many studies. Here, indicate whether each material, system or method listed is relevant to your study. If you are not sure if a list item applies to your research, read the appropriate section before selecting a response.

Materials & experimental systems

n/a	Involved in the study
☒	☐ Antibodies
☒	☐ Eukaryotic cell lines
☒	☐ Palaeontology and archaeology
☒	☐ Animals and other organisms
☒	☐ Clinical data
☒	☐ Dual use research of concern
☒	☐ Plants

Methods

n/a	Involved in the study
☒	☐ ChIP-seq
☒	☐ Flow cytometry
☒	☐ MRI-based neuroimaging
